# CD28 superagonist-mediated boost of regulatory T cells increases thrombo-inflammation and ischemic neurodegeneration during the acute phase of experimental stroke

**DOI:** 10.1038/jcbfm.2014.175

**Published:** 2014-10-15

**Authors:** Michael K Schuhmann, Peter Kraft, Guido Stoll, Kristina Lorenz, Sven G Meuth, Heinz Wiendl, Bernhard Nieswandt, Tim Sparwasser, Niklas Beyersdorf, Thomas Kerkau, Christoph Kleinschnitz

**Affiliations:** 1 Department of Neurology, University Clinics Würzburg, Würzburg, Germany; 2 Institute of Clinical Epidemiology and Biometry, Comprehensive Heart Failure Center, University of Würzburg, Würzburg, Germany; 3 Institute of Pharmacology and Toxicology, University of Würzburg, Würzburg, Germany; 4 Department of Neurology, University of Münster, Münster, Germany; 5 Institute of Physiology I—Neuropathophysiology, University of Münster, Münster, Germany; 6 University Hospital and Rudolf Virchow Center, University of Würzburg, Würzburg, Germany; 7 Institute of Infection Immunology, TWINCORE, Centre for Experimental and Clinical Infection Research; a joint venture between the Medical School Hannover and the Helmholtz Centre for Infection Research, Hannover, Germany; 8 Institute for Virology and Immunobiology, University of Würzburg, Würzburg, Germany

**Keywords:** brain ischemia, inflammation, microcirculation, T cells, thrombosis

## Abstract

While the detrimental role of non-regulatory T cells in ischemic stroke is meanwhile unequivocally recognized, there are controversies about the properties of regulatory T cells (T_reg_). The aim of this study was to elucidate the role of T_reg_ by applying superagonistic anti-CD28 antibody expansion of T_reg_. Stroke outcome, thrombus formation, and brain-infiltrating cells were determined on day 1 after transient middle cerebral artery occlusion. Antibody-mediated expansion of T_reg_ enhanced stroke size and worsened functional outcome. Mechanistically, T_reg_ increased thrombus formation in the cerebral microvasculature. These findings confirm that T_reg_ promote thrombo-inflammatory lesion growth during the acute stage of ischemic stroke.

## Introduction

It has been observed for many years that immune cells transmigrate over the blood–brain barrier into infarcted brain tissue. This process follows a defined time course, with neutrophils and macrophages beginning to accumulate in the brain some hours after stroke, and lymphocytes reaching maximum infiltration by approximately day 3.^1^ While the detrimental role of non-regulatory T-cell subsets in the acute phase of ischemic stroke is widely accepted,^2, 3, 4^ there is an ongoing debate about the contribution of regulatory T cells (T_reg_) to this pathology.^4, 5, 6, 7^

We were able to show that T_reg_ cause microvascular dysfunction and consequently thrombus formation and secondary infarct growth in the acute phase after experimental ischemic stroke by interaction with endothelial cells and platelets.^4,8^ This interplay between inflammatory processes and thrombus formation has recently been referred to as ‘thrombo-inflammation'.^8^

Other groups reported secondary deterioration of stroke volumes and functional outcome in animals 7 days after blocking of T_reg_.^5^ Therefore, the aim of this study was to clarify the impact of T_reg_ in the acute phase of ischemic stroke by the means of a superagonistic anti-CD28 antibody (CD28 SA) that leads to an expansion of pre-existing T_reg_ in the lymphoid organs and the dissemination of increased T_reg_ numbers in the peripheral blood.^9,10^

## Materials and Methods

Details of the experimental procedure are provided in the Supplementary Information. The animal experiments were conducted in accordance with the recommendations of the European Convention for the Protection of Vertebrate Animals used for Experimentation and the current ARRIVE guidelines (http://www.nc3rs.org/ARRIVE). Experiments were approved by legal state authorities (Government of Lower Franconia).

### Ischemia Model

Focal cerebral ischemia was induced in 6 to 8-week-old male C57BL/6, DEREG or *Rag1*^*−/−*^ mice by 30-minute transient middle cerebral artery occlusion (tMCAO) as described.^3,4,11^ Infarct volumes were calculated from brain slices stained with 2,3,5-triphenyltetrazolium chloride. The Bederson score and the grip test score were used to monitor neurologic function.^12,13^

### Expansion of T_reg_ by CD28 SA Treatment *in vivo*

CD28 SA (clone D665, Exbio, Praha, Czech Republic, 50 *μ*g/mouse) was applied 3 days before tMCAO (prophylactic application) or immediately after tMCAO (therapeutic application) by an intraperitoneal injection. MOPC-21 antibody (BioXCell, West Lebanon, NH, USA) served as control. Regulatory T cells were quantified by flow cytometry analysis (Foxp3 staining). Depletion of T_reg_ in DEREG mice was achieved by the administration of diphtheria toxin (DTX, Merck, Darmstadt, Germany, 1 *μ*g/mouse per day intraperitoneally) on three consecutive days before tMCAO.

### Immunohistochemistry

Cryoembedded brain slices were stained with antibodies against CD31 (Abcam, ab9498, Cambridge, UK), CD4 (100506, BioLegend, San Diego, CA, USA), Ly6B.2 (MCA771GA, Serotec, Puchheim, Germany) or AF488-conjugated anti-platelet glycoprotein IX (generated by B Nieswandt, Würzburg, Germany). For quantification of occluded vessels hematoxylin–eosin staining was performed (10 *μ*m slices), and the percentage of occluded vessels in every tenth slice was counted under 40-fold magnification.

## Results

First, we confirmed that CD28 SA treatment leads to an expansion of T_reg_ in mice (maximum on day 3), as has previously been described.^10^ We found a significant increase in the relative numbers of T_reg_ in the blood and lymph nodes of wild-type mice (Supplementary Figure 1, *P*<0.05). In the next step, we ruled out that the CD28 SA itself influences the cerebral blood flow, hemodynamics (heart rate and mean arterial pressure) and the blood gas analysis (Supplementary Figures 2 and 3, Supplementary Table 1). These findings exclude that CD28 SA treatment alters important physiologic parameters that might influence stroke outcome and prove that middle cerebral artery occlusion and reperfusion were sufficient in our model.

**Figure 1:**
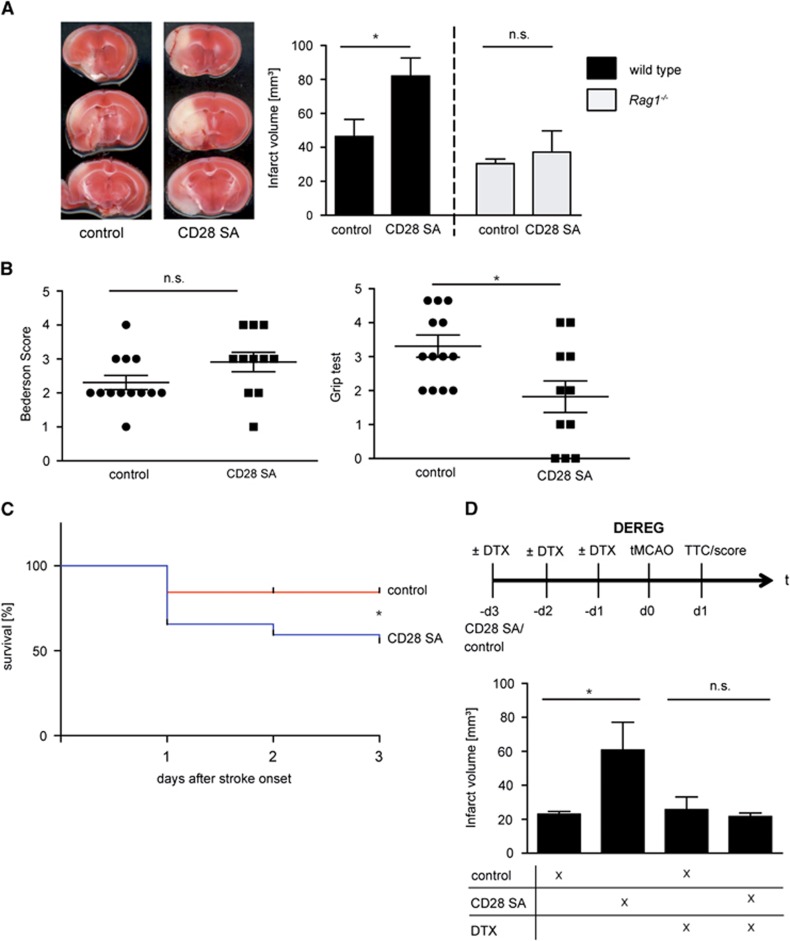
Increase in superagonistic anti-CD28 antibody (CD28 SA)-induced T_reg_ worsens stroke outcome. (**A**) Left panel: representative 2,3,5-triphenyltetrazolium chloride stains of coronal brain sections of control and CD28 SA-treated wild-type mice on day 1 after transient middle cerebral artery occlusion (tMCAO). Right panel: infarct volume was increased in the CD28 SA-treated group (*n*=11–13 per group) in wild-type animals, but not in *Rag1*^*−/−*^ mice (*n*=7 or 8 per group), unpaired, two-tailed Student's *t*-test. (**B**) CD28 SA-treated mice only had a slightly worse Bederson score (left panel) but performed significantly worse in the gript test (right panel) (*n*=11–13 per group), Mann–Whitney test. (**C**) Analysis of survival using a Kaplan–Meier curve (plotted from four independent experiments with each experiment including five mice per group), Log-rank test. (**D**) CD28 SA induces larger infarctions in DEREG mice still containing T_reg_, i.e., without diphtheria toxin (DTX) pretreatment, compared with mice receiving control antibody. In contrast, CD28 SA was unable to enhance lesion size in DEREG mice devoid of T_reg_, i.e., after DTX pretreatment (*n*=5–6 per group), one-way analysis of variance with *post hoc* Bonferroni adjustment for *P* values. NS=not significant; *P<0.05.

Next, we assessed if the increase in T_reg_ numbers before tMCAO influences stroke development in wild-type mice. Stroke volumes on day 1 were significantly larger (82.0±35.2 mm^3^) compared with wild-type animals that had received isotype control antibodies (46.5±36.4 mm^3^, *P*<0.05) (Figure 1A). To exclude that the CD28 SA influences outcome measures independent of its boosting effect on the T_reg_ population, we additionally analyzed *Rag1*^*−/−*^ mice that lack lymphocytes. As the CD28 SA had no impact on stroke volumes (control group: 30.4±2.8 mm^3^; CD28 SA group: 37.3±12.5 mm^3^, *P*>0.05) (Figure 1A) a direct effect of the CD28 SA on clinical outcome in the absence of T and B cells could be excluded. Importantly, increased stroke size in the wild-type mice also translated into worse functional outcome as assessed by the grip test (values are the median with 25th and 75th percentiles, respectively, in brackets (control: 3.0 (2.0, 4.5); CD28 SA: 2.0 (0.0, 3.0), *P*<0.05), but not in the Bederson score (control: 2.0 (2.0, 3.0); CD28 SA: 3.0 (2.0, 4.0), *P*>0.05) (Figure 1B). Again, there was no difference regarding behavioral testing between the treatment groups in *Rag1*^*−/−*^ animals (not shown). Moreover, survival rates on days 1 and 3 were lower in CD28 SA pretreated animals (Figure 1C). Importantly, treatment with CD28 SA after tMCAO (therapeutic approach) had no impact on stroke volumes (Supplementary Figure 4) and functional outcome (not shown) on day 3.

*Rag1*^*−/−*^ mice do not exclusively lack T_reg_. To rule out the fact that the detrimental effect of the CD28 SA was at a relevant portion mediated by other lymphocyte subpopulations apart from T_reg_, we introduced the DEREG mouse model. In these mice T_reg_ can be selectively depleted by the repetitive application of DTX.^11^ Superagonistic anti-CD28 antibody again significantly increased the numbers of circulating T_reg_ in naïve DEREG mice but not in T_reg_-depleted DEREG mice (Supplementary Figure 5). Accordingly, CD28 SA pretreatment induced significantly larger infarctions in DEREG mice containing T_reg_, while this was no longer the case in the group of DEREG mice without T_reg_ (Figure 1D).

**Figure 2:**
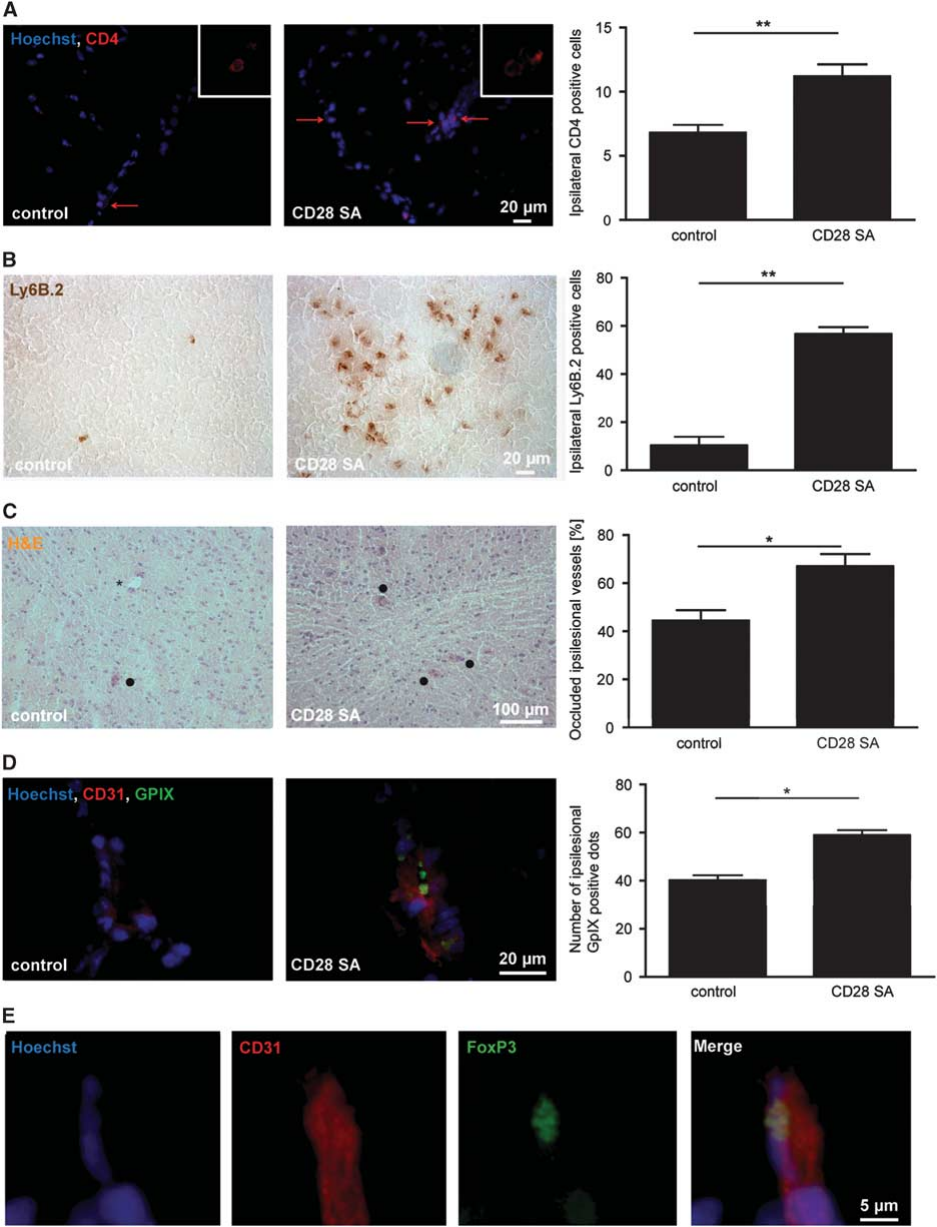
CD28 SA treatment increases intracerebral immune cell accumulation and thrombosis after transient middle cerebral artery occlusion (tMCAO). (**A**) Representative immunocytologic stainings of brain-infiltrating CD4^+^ T lymphocytes (indicated by red arrows) on day 1 after tMCAO. Quantification revealed significantly higher numbers of CD4^+^ T lymphocytes in the ipsilesional hemispheres of superagonistic anti-CD28 antibody (CD28 SA)-treated mice when compared with MOPC-21 (control) treated mice (*n*=5 per group). **P<0.01, unpaired, two-tailed Student's *t*-test. (**B**) Representative immunocytologic stainings of brain-infiltrating Ly6B.2^+^ neutrophils on day 1 after tMCAO. Quantification also revealed significantly higher numbers of Ly6B.2^+^ neutrophils in the ipsilesional hemispheres of CD28 SA-treated mice when compared with MOPC-21 (control) treated mice (*n*=3 per group), ^**^P<0.01, unpaired, two-tailed Student's *t*-test. (**C**) Representative hematoxylin–eosin (H&E)-stained brain sections on day 1 after tMCAO. Quantification of occluded ipsilesional vessels (black dots) revealed a significant increase in the CD28 SA-treated group (*n*=4 per group), *P<0.05, unpaired, two-tailed Student's *t*-test. The asterisk indicates a patent microvessel. (**D**) Representative immunocytologic stainings of platelet aggregates within the vasculature on day 1 after tMCAO. Hoechst depicts cell nuclei, CD31 stains endothelial cells. Quantification revealed significantly higher numbers of ipsilesional glycoprotein IX (GPIX)-positive aggregates in CD28 SA-treated mice when compared with control mice. (*n*=4 per group), *P<0.05, unpaired, two-tailed Student's *t*-test. (**E**) Immunohistochemical staining of brain sections from DEREG mice on day 1 after 30 minutes of tMCAO showing green fluorescent protein-positive Foxp3^+^ T_reg_ predominantly in the cerebral vasculature (co-stained with CD31).

In the next step, we analyzed if the increase in peripheral T_reg_ alters the composition of the cellular infiltrate within the ischemic brain. Indeed, we found more ipsilesional CD4^+^ cells (Figure 2A, *P*<0.01) and neutrophils (Figure 2B, *P*<0.01) after pre-tMCAO CD28 SA treatment compared with control animals. A negative immunostaining control is provided in Supplementary Figure 6. The fact that CD28 SA treatment not only increased the amount but also the density of immune cells (Supplementary Figure 7) argues against an unspecific effect, related for instance to larger infarct volumes. Nevertheless, data must be interpreted with caution since brains have not been flushed before sampling in these experiments. Hence, the total cell numbers obtained here potentially include a considerable amount of cells derived from within the brain vasculature.

To confirm that T_reg_ contribute to ischemic brain injury by boosting ‘thrombo-inflammation',^8^ we demonstrated a higher number of occluded brain vessels (Figure 2C, *P*<0.05) and glycoprotein IX-positive platelets (Figure 2D, *P*<0.05) in CD28 SA-pretreated mice compared with control mice. Again, the CD28 SA also increased the density of occluded vessels (Supplementary Figure 8). To further analyze the location of T_reg_ in the ischemic brain during the early phase of stroke, we performed immunohistochemistry of brain specimen taking advantage of genetically modified mice, in which Foxp3-expressing cells are visible by a transgenic construct linking green fluoresecent protein and the DTX receptor (DEREG mice).^4,11^ We could show that T_reg_ were predominantly located within the vessel lumina (Figure 2E) and did not transmigrate into the brain parenchyma until day 1 after stroke, which is in line with previous studies.^1,4^

## Discussion

In the present study, with the use of a pharmacologic expansion of T_reg_, we independently confirm that T_reg_ are strong potentiators of acute ischemic stroke.^4^ Superagonistic anti-CD28 antibody-induced expansion of T_reg_ positively correlated with increased stroke size 24 hours after ischemia. In analogy to our previous study, T_reg_ interact with endothelial cells and platelets to induce microvascular dysfunction and thrombosis.^4^ In contrast to our ancestor study^4^ and the results of this study—both strongly arguing for a detrimental role of T_reg_ during the acute phase of brain ischemia/reperfusion injury—it has been reported that T_reg_ are key modulators of cerebroprotection in brain ischemia in mice in the late phase.^5^ Therefore, it would be worthwhile to study whether CD28 SA application at later time points influences stroke outcome although T_reg_ expansion with CD28 SA after tMCAO had no impact on the neurological status on day 3 in our hands (Supplementary Figure 4).

T_reg_ promote stroke progression within a few hours after cessation of cerebral blood flow. At this early stage, they are mainly found within cerebral blood vessels and interact with endothelial cells and platelets. This interplay between thrombotic and inflammatory processes has recently been described as ‘thrombo-inflammation'.^8^ Nevertheless, we also found an increased number of CD4^+^ and Ly6B.2^+^ cells in the brain parenchyma as early as day 1 after tMCAO. While it is well-known that neutrophils start to transmigrate into the brain within hours after stroke, the pathophysiologic role of these cells in acute ischemic stroke is still under debate.^1,4,14^ Moreover, the mechanisms through which peripheral T_reg_ expansion by the CD28 SA enhances neutrophil recruitment to the ischemic brain need to be further established. One potential explanation could include the formation of a pro-inflammatory milieu triggered by activated T cells present in high numbers within the brain vasculature and subsequent upregulation of cell adhesion molecules. However, based on the observation that T_reg_ (and other T cells) trigger ischemic brain damage already after a few hours after vessel occlusion, it can be assumed that mechanisms operating mainly within the brain-perfusing vessels rather than within the brain parenchyma contribute to the detrimental T-cell effect to a large extent.^2, 3, 4^

In summary, the present study independently confirms that T_reg_ promote lesion growth during the acute stage of ischemic stroke. Short-term inhibition of T_reg_ might become a promising therapeutic approach to combat this devastating neurologic condition.
