# Supplementary material for: CD28 superagonist-mediated boost of regulatory T cells increases thrombo-inflammation and ischemic neurodegeneration during the acute phase of experimental stroke
Source: J Cereb Blood Flow Metab. 2014 Oct 15;35(1):6–10. doi: 10.1038/jcbfm.2014.175 (PMC4294400; doi:10.1038/jcbfm.2014.175)
Supplement: Supplementary Information [file jcbfm2014175x1.doc]

**ONLINE SUPPLEMENT**

**CD28 superagonist-mediated boost of regulatory T cells increases thrombo-inflammation and ischemic neurodegeneration during the acute phase of experimental stroke**

Michael K Schuhmann, PhD^1,*^, Peter Kraft, MD^1,2,*^, Guido Stoll, MD^1^, Kristina Lorenz, PhD^3^, Sven G Meuth, MD, PhD^4,5^, Heinz Wiendl, MD^4^, Bernhard Nieswandt, PhD^6^, Tim Sparwasser, MD^7^, Niklas Beyersdorf, MD^8^, Thomas Kerkau, MD^8^, Christoph Kleinschnitz, MD^1^

**Supplemental Methods**

**Mice, Stroke Model and Animal Treatment**

A total of 116 C57BL/6, 31 DEREG and 15 *Rag1^−/−^* mice were used in this study. Animal experiments were approved by legal state authorities (Government of Lower Franconia) and conducted in accordance with the recommendations for research in experimental stroke studies[^1^](#_ENREF_1) and the current ARRIVE guidelines (http://www.nc3rs.org/ARRIVE). Mice were randomly assigned to the operators by an independent person not involved in data analysis. We performed surgery and evaluation of all readout parameters while being blinded to the experimental groups. Focal cerebral ischemia was induced in 6–8-week-old male mice (Harlan Winkelmann, Darmstadt, Germany) by 30-min transient middle cerebral artery occlusion (tMCAO) as previously described.[^2^](#_ENREF_2) Mice were anesthetized with 2.5% isoflurane (Abbott, Wiesbaden, Germany). Following a midline skin incision in the neck, the proximal common carotid artery and the external carotid artery were ligated and a standardized silicon rubber-coated 6.0 nylon monofilament (60SPPK10; Doccol Corp.) was inserted and advanced via the right internal carotid artery to occlude the origin of the right middle cerebral artery (MCA). The operators were blinded to the treatment groups and the maximum operation time per animal did not exceed 15 min. Three days before tMCAO or immediately after the removal of the filament and MCA reperfusion, the CD28 superagonist (SA) (clone D665, Exbio, Praha, Czech Republic, 50 µg/mouse) was applied by a single intraperitoneal (i.p.) injection. The MOPC-21 antibody (BioXCell, West Lebanon, United States) served as isotype control.

The following conditions excluded mice from end-point analyses (exclusion criteria):[^3^](#_ENREF_3)

1. Death within 24 hours after tMCAO

2. Subarachnoid hemorrhage or bleeding into the brain parenchyma (as macroscopically assessed during brain sampling)

3. Bederson score = 0 (immediately after reperfusion)

4. Operation time > 15 minutes

**Determination of Stroke Size**

Animals were sacrificed 24 hours after tMCAO. Brains were removed (without previous flushing) and cut into three 2-mm-thick coronal slices using a mouse brain slice matrix (Harvard Apparatus). The sections were stained with 2,3,5-triphenyltetrazoliumchloride for 20 min at 37°C and edema-corrected infarct volumes were quantified by planimetry (ImageJ software, National institutes of health) according to the following equation:

*V*_indirect_(mm^3^) = *V*_infarct_ × (1−(*V*_I_ − *V*_C_)/*V*_C_),

with the term (*V*_I_ − *V*_C_) representing the volume difference between the ischemic hemisphere and the control hemisphere and (*V*_I_ − *V*_C_)/*V*_C_) expressing this difference as a percentage of the control hemisphere.

**Assessment of Functional Outcome**

Twenty-four hours after tMCAO, the modified Bederson score[^4^](#_ENREF_4) was used to determine global neurologic function according to the following scoring system: 0, no deficit; 1, forelimb flexion; 2, decreased resistance to lateral push; 3, unidirectional circling; 4, longitudinal spinning; 5, no movement. Motor function and coordination were evaluated by the grip test.[^5^](#_ENREF_5) For this test, the mouse was placed midway on a string between two supports and rated as follows: 0, falls off; 1, hangs onto string by one or both forepaws; 2, as for 1, and attempts to climb onto string; 3, hangs onto string by one or both forepaws plus one or both hindpaws; 4, hangs onto string by fore- and hindpaws plus tail wrapped around string; 5, escape (from the supports). Neurologic scores were assessed by an independent investigator blinded to the experimental conditions of the mice.

**Laser-Doppler Flowmetry**

Laser-Doppler flowmetry (Moore Instruments) was performed in isotype- and CD28 SA-treated wild-type mice before (baseline), during (ischemia) and immediately after tMCAO (reperfusion). The regional cerebral blood flow was measured in the area of the right MCA (6 mm lateral and 2 mm posterior from bregma).[^6^](#_ENREF_6)

**Invasive Hemodynamics**

For the assessment of blood pressure and heart rate, isotype- and CD28 SA-treated wild-type mice were anesthetized with 2.0% isoflurane and catheterized via the right carotid artery with a high-fidelity 1.4 F Millar microtip catheter (Milar Instruments) as described.[^7^](#_ENREF_7)

**Blood Gas Analysis**

Arterial blood (100 μL) was drawn from the left cardiac ventricle of anesthetized mice by a heparinized syringe. We determined PaO_2_, PaCO_2_ and pH in isotype- and CD28 SA-treated mice using an ABL 77 automated blood gas analyzer (Radiometer).

**Immunohistochemistry and Histology**

Cryoembedded brains were cut into 10-µm-thick sections on day 1 after tMCAO. For immunofluorescence stainings, we pretreated the sections with 10% bovine serum albumin (BSA) and 1% goat serum with 0.2% Triton X-100 for 30 min to prevent unspecific binding. For specific staining, the following antibodies were added overnight at 4°C: CD31 (mouse anti-mouse, Abcam, ab9498, 1:100), CD4 (BioLegend, 100506, 1:100) or AF488-GPIX (rat anti-mouse, emfret Analytics, 1:100). Subsequently, slices were incubated with Dylight488-coupled goat anti-mouse (Abcam, 96871, 1:100) and AlexaFluor594 goat anti-rat (Invitrogen, A11012, 1:100) antibodies in phosphate-buffered saline (PBS) containing 1% BSA. For staining of neutrophils Ly-6B.2 (rat anti-mouse, MCA771G, AbD Serotec, 1:500) was added in PBS containing 1% BSA overnight at 4°C. Afterwards, slides were incubated with a biotinylated anti-rat IgG (BA-4001, Vector Laboratories, 1:100) in PBS containing 1% BSA for 45 min at room temperature. Following treatment with Avidin/Biotin blocking solution (Avidin/Biotin Blocking Kit, Sp-2001, Vector Laboratories) to inhibit endogenous peroxidase activity, the secondary antibody was linked via streptavidin to a biotinylated peroxidase (POD) according to the manufacturer’s instructions (Vectorstain ABC Kit, Peroxidase Standard PK-4000, Vector Laboratories). Antigens were visualized via POD using the chromogen 3,3′- diaminobenzidine (Kem-En-Tec Diagnostics). Sections were analyzed under a microscope (Nikon Eclipse 50i) equipped with a charge-coupled device camera. We analyzed 4-5 slices per animal and counted the total number of infiltrated cells per ipsilesional hemisphere using a 20 or 40 fold magnification. Negative controls included omission of primary or secondary antibody and gave no signals (Supplementary Figure 6).

For calculation of the thrombosis index, the whole brain was sliced 24 hours after tMCAO (slice thickness 10 µm). Hematoxylin–eosin staining was performed according to standard procedures. For quantification, stainings were examined in a blinded fashion and the percentage of occluded blood vessels within the ischemic hemispheres was counted in every tenth slice for CD28 SA-treated and isotype control-treated animals under 40-fold magnification.

For quantification of the density of CD4+ and Ly6B.2+ cells as well as the occluded vessels we analyzed 4 optical fields per slice and brain region of 4-5 slices per animal using 20-fold (Ly6B.2 and H&E staining) or 40-fold (CD4 staining) magnification.

**Cell Separation and Flow Cytometry**

For flow cytometry analysis of peripheral immune cells, 100 μl blood (+ lymph nodes) was harvested transcardially in heparin-coated tubes and red blood cells were lysed using RBC lysis buffer (BioLegend) following the manufacturer’s instructions. CD4^+^ (anti-CD4-APC, BioLegend, 100412) and FoxP3^+^ (anti-FoxP3-PE, BioLegend, 79474) cells were stained using a FoxP3-staining kit (BioLegend) according to the manufacturer’s instructions. Flow cytometry was performed using a FACSCalibur (Becton Dickinson).

**Statistics**

All data are given as mean ± SEM except for the ordinal Bederson score and the grip-test score which are depicted as scatter plots including median with the 25th percentile and the 75th percentile given in brackets in the text. Numbers of animals (*n* = 10) necessary to detect a standardized effect size on infarct volumes ≥ 0.25 (CD28 SA-treated versus isotype-treated mice) were calculated via *a priori* sample size analysis with the following assumptions: α = 0.05, β = 0.2 (power 80%), mean, standard deviation 20% of the mean (StatMate 2.0, GraphPad Software). For statistical analysis, PrismGraph 5.0 software package (GraphPad Software) was used. Data were tested for Gaussian distribution with the Kolmogorov–Smirnov test and then analyzed by the unpaired, two-tailed Student *t*-test or the non-parametric Mann–Whitney test (Bederson score and the grip test). 1-way ANOVA with post hoc Bonferroni correction was applied when comparing more than two groups. Survival was plotted as Kaplan Meier curve and compared using the log-rank test. *P* < 0.05 was considered statistically significant.

**Supplementary References**

1. Dirnagl U. Bench to bedside: The quest for quality in experimental stroke research. *J Cereb Blood Flow Metab* 2006; **26**: 1465-1478.

2. Kleinschnitz C, Kraft P, Dreykluft A, Hagedorn I, Gobel K, Schuhmann MK *et al.* Regulatory T cells are strong promoters of acute ischemic stroke in mice by inducing dysfunction of the cerebral microvasculature. *Blood* 2013; **121**: 679-691.

3. Kraft P, Gob E, Schuhmann MK, Gobel K, Deppermann C, Thielmann I *et al.* FTY720 ameliorates acute ischemic stroke in mice by reducing thrombo-inflammation but not by direct neuroprotection. *Stroke* 2013; **44**: 3202-3210.

4. Bederson JB, Pitts LH, Tsuji M, Nishimura MC, Davis RL, Bartkowski H. Rat middle cerebral artery occlusion: Evaluation of the model and development of a neurologic examination. *Stroke* 1986; **17**: 472-476.

5. Moran PM, Higgins LS, Cordell B, Moser PC. Age-related learning deficits in transgenic mice expressing the 751-amino acid isoform of human beta-amyloid precursor protein. *Proc Natl Acad Sci U S A* 1995; **92**: 5341-5345.

6. Connolly ES, Jr., Winfree CJ, Springer TA, Naka Y, Liao H, Yan SD *et al.* Cerebral protection in homozygous null icam-1 mice after middle cerebral artery occlusion. Role of neutrophil adhesion in the pathogenesis of stroke. *J Clin Invest* 1996; **97**: 209-216.

7. Brede M, Braeuninger S, Langhauser F, Hein L, Roewer N, Stoll G *et al.* Alpha(2)-adrenoceptors do not mediate neuroprotection in acute ischemic stroke in mice. *J Cereb Blood Flow Metab* 2011; **31**: e1-7.

**Supplemental Figures**


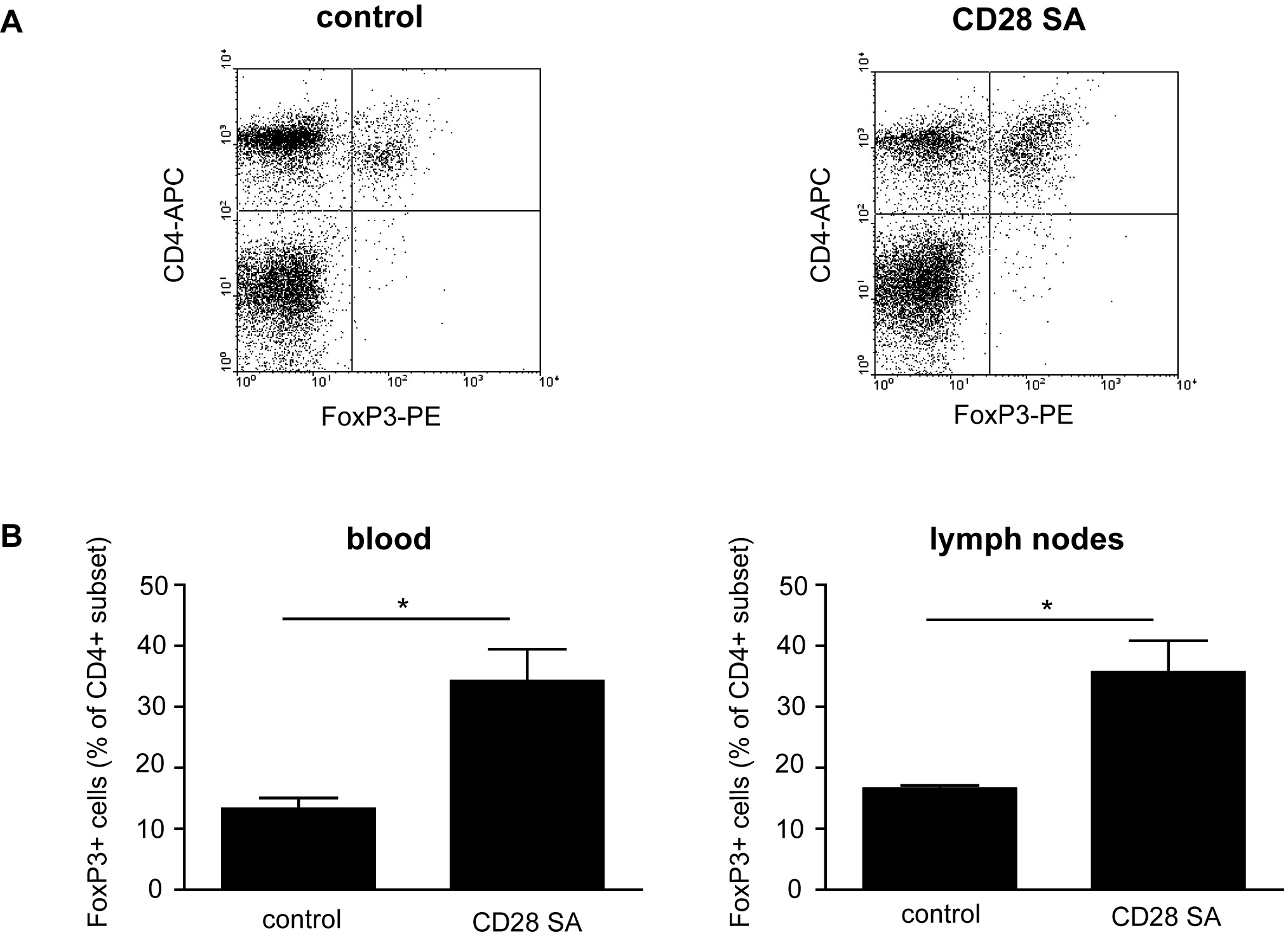


**Supplemental Figure 1**. CD28 SA induces a rapid increase of T_reg_ in the peripheral blood and lymph nodes. (**A**) Visualization (dot plot) of FoxP3^+^ T_reg_ from lymph nodes 3 days after treatment with control (left) and CD28 SA (right) assessed by flow cytometry. (**B**) Fraction of FoxP3^+^ T_reg_ from the total CD4^+^ cell number 3 days after treatment with control or CD28 SA. *N* = 4 or 5 per group, **P* < 0.05, unpaired, two-tailed Student *t*-test. CD28 SA, cluster of differentiation 28 superagonist.

**Supplemental Figure 2.** CD28 SA does not change regional cerebral blood flow (rCBF). Determination of rCBF using Laser Doppler flowmetry before (baseline), 10 min after the occlusion of the middle cerebral artery (ischemia) and again 10 min after the removal of the occluding filament (reperfusion) in C57BL/6 mice treated with isotype control or CD28 SA. No significant differences in rCBF were observed between the groups. *n* = 3. CD28 SA, cluster of differentiation 28 superagonist.

**
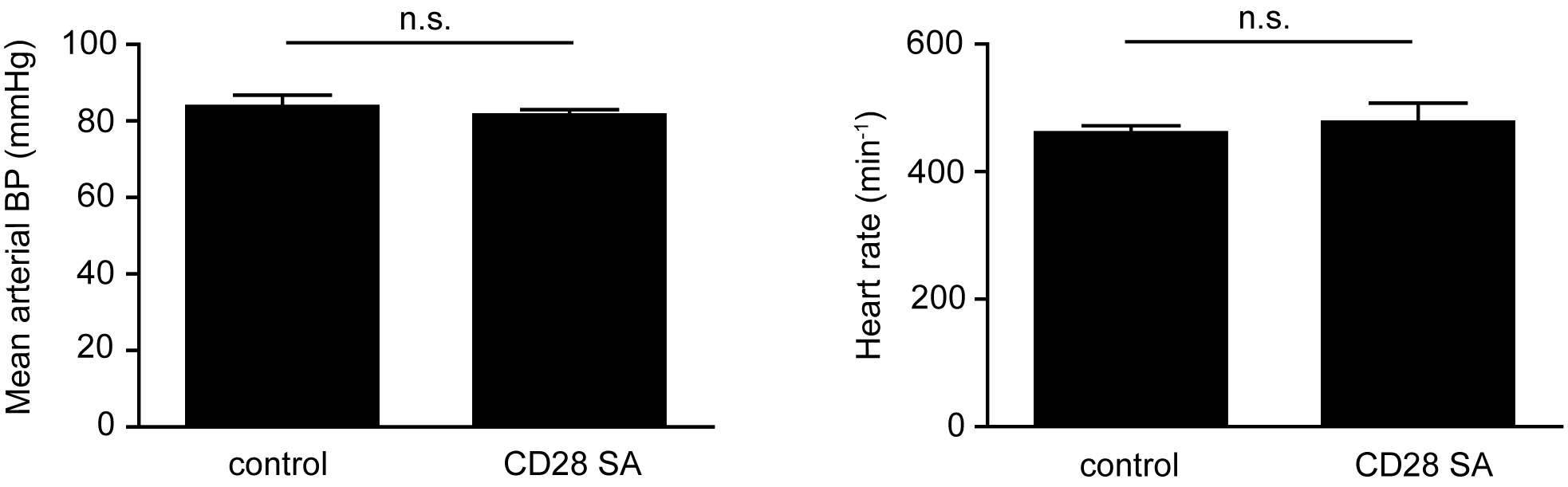
**

**Supplemental Figure 3.** CD28 SA does not change hemodynamics. Mean arterial blood pressure (BP; left) and heart rate (right) are unaltered in isotype- and CD28 SA-treated wild-type mice as assessed by invasive hemodynamics. *n* = 4 or 5 per group. Unpaired, two-tailed Student *t*-test. CD28 SA, cluster of differentiation 28 superagonist; n.s., not significant.


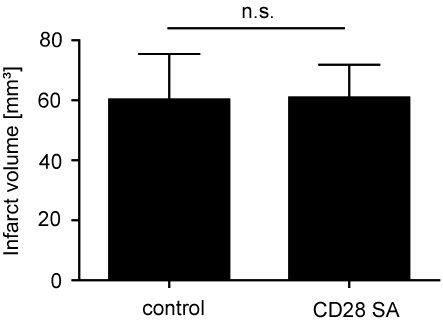


**Supplemental Figure 4.** Therapeutical administration of CD28 SA had no influence on stroke volumes. CD28 SA or isotype control were given immediately after reperfusion. Stroke volumes were assessed at day 3 using TTC staining. n = 7 per group. Unpaired, two-tailed Student *t*-test. CD28 SA, cluster of differentiation 28 superagonist; n.s., not significant.


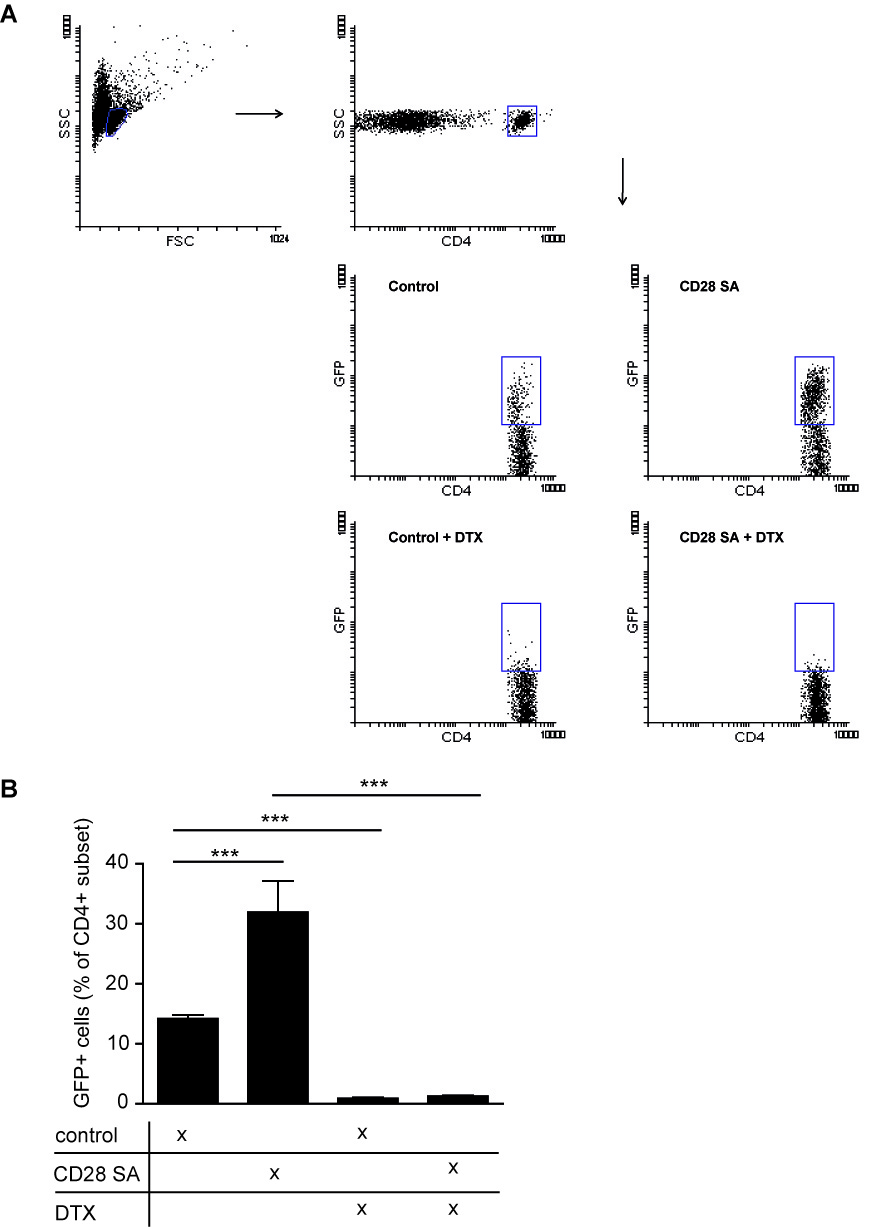


**Supplemental Figure 5**. CD28 SA induces an increase of GFP positive T_reg_ in lymph nodes from naïve DEREG mice but not in Treg-depleted DEREG mice. (**A**) Visualization (dot plot) of the gating strategy and representative treatment groups as assessed by flow cytometry. (**B**) Quantification of the fraction of GFP+ T_reg_ from the total CD4^+^ cell number 1 day after tMCAO (n ≥ 4 per group), ****P* < 0.005, unpaired, two-tailed Student *t*-test. CD28 SA, cluster of differentiation 28 superagonist; DTX, diphtheria toxin.


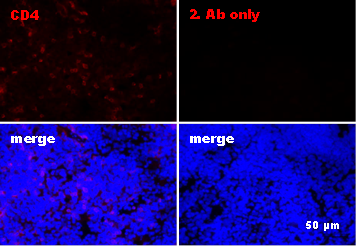


50 µm

**Supplemental Figure 6.** Positive and negative staining controls for CD4 stainings derived from spleen (20 fold magnification). Ab, antibody.


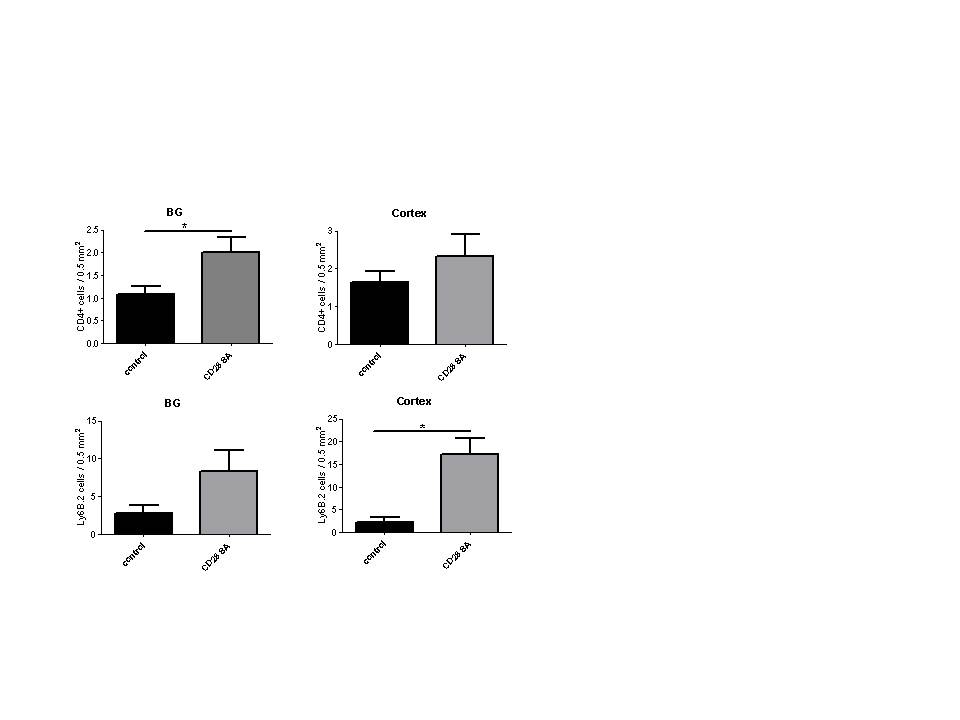


**Supplemental Figure 7.** Density of CD4 (upper panel) and Ly6B.2 positive cells (lower panel) per 0.5 mm² brain tissue as assessed with 20 (Ly6B.2) or 40 fold (CD4) magnification. Four fields per slice and region have been counted. **P* < 0.05, unpaired, two-tailed Student *t*-test. CD28 SA, cluster of differentiation 28 superagonist; BG, basal ganglia.

**Supplemental Figure 8.** Density of occluded vessels per mm² brain tissue as assessed with 20 fold magnification. Four fields per slice and region have been counted. **P* < 0.05, unpaired, two-tailed Student *t*-test. CD28 SA, cluster of differentiation 28 superagonist; BG, basal ganglia.

|  | **Isotype control** | **CD28 SA** | ***P*** |
| --- | --- | --- | --- |
| **n** | 3 | 3 | n.s. |
| **PaO_2_ (mmHg)** | 71.7 ± 6.7 | 79.0 ± 9.6 | n.s. |
| **PaCO_2_ (mmHg)** | 40.3 ± 2.1 | 39.0 ± 3.0 | n.s. |
| **pH** | 7.30 ± 0.05 | 7.34 ± 0.06 | n.s. |

**Supplemental Table 1.** Blood gas analysis in isotype- or CD28 SA-treated wild-type mice. No significant differences were observed between the groups. Unpaired, two-tailed Student *t*-test. n.s., not significant; CD28 SA, cluster of differentiation 28 superagonist.
